# Cilia at the crossroad: convergence of regulatory mechanisms to govern cilia dynamics during cell signaling and the cell cycle

**DOI:** 10.1186/s13578-025-01403-z

**Published:** 2025-06-07

**Authors:** Lukáš Čajánek, Sindija Smite, Olha Ivashchenko, Martina Huranova

**Affiliations:** 1 Laboratory of Cilia and Centrosome Biology, Department of Histology and Embryology, Faculty of Medicine, Masaryk University, Kamenice 3, Brno, 62500 Czech Republic; 2 Section of Animal Physiology and Immunology, Department of Experimental Biology, Faculty of Science, Masaryk University, Kamenice 5, Brno, 62500 Czech Republic; 3 Laboratory of Cilia Genetics and Pathology, Institute of Molecular Genetics of the Czech Academy of Sciences, Vídeňská 1083, Prague, 142 00 Czech Republic

**Keywords:** Cilia, Ciliary dynamics, Ciliary signaling, Cell cycle regulation, Tissue development, Ciliopathies, Cancer

## Abstract

Cilia are versatile, microtubule-based organelles that facilitate cellular signaling, motility, and environmental sensing in eukaryotic cells. These dynamic structures act as hubs for key developmental signaling pathways, while their assembly and disassembly are intricately regulated along cell cycle transitions. Recent findings show that factors regulating ciliogenesis and cilia dynamics often integrate their roles across other cellular processes, including cell cycle regulation, cytoskeletal organization, and intracellular trafficking, ensuring multilevel crosstalk of mechanisms controlling organogenesis. Disruptions in these shared regulators lead to broad defects associated with both ciliopathies and cancer. This review explores the crosstalk of regulatory mechanisms governing cilia assembly, disassembly, and maintenance during ciliary signaling and the cell cycle, along with the broader implications for development, tissue homeostasis, and disease.

## Current model of primary cilia assembly and disassembly

Cilia are hair-like organelles composed of microtubules that extend from the cell surface of most eukaryotic cells. Cilia are probably the first ever observed cellular organelles, already described in the 17th century by a Dutch pioneer of microbiology and microscopy, Antonie van Leeuwenhoek [1]. Cilia can be motile or non-motile, with non-motile cilia referred to as primary cilia. The “primary” aspect of primary cilia relates to the time of their appearance, as primary cilium was noted to appear before the motile cilia in cells of rat lung epithelium [2]. Initially, primary cilia were thought to be vestigial, owing to their lack of motility. However, research over the past two decades has significantly twisted this perspective, revealing that primary cilia serve as crucial cellular antennas with essential roles in embryogenesis and tissue homeostasis [3, 4], [5]. Cilia significance has become even more evident with the discovery that a variety of human diseases, collectively termed ciliopathies, are linked to defects in ciliary structure and/or function [6, 7, 8]. In our review, we discuss primary cilia as dynamic organelles responding to various regulatory clues during their assembly, disassembly, and maintenance and, in turn, how the dynamic nature of primary cilia influences fundamental biological processes, including cell fate determination, tissue patterning, signal transduction, and cellular homeostasis.

### Primary cilia and their structure

**Fig. 1:**
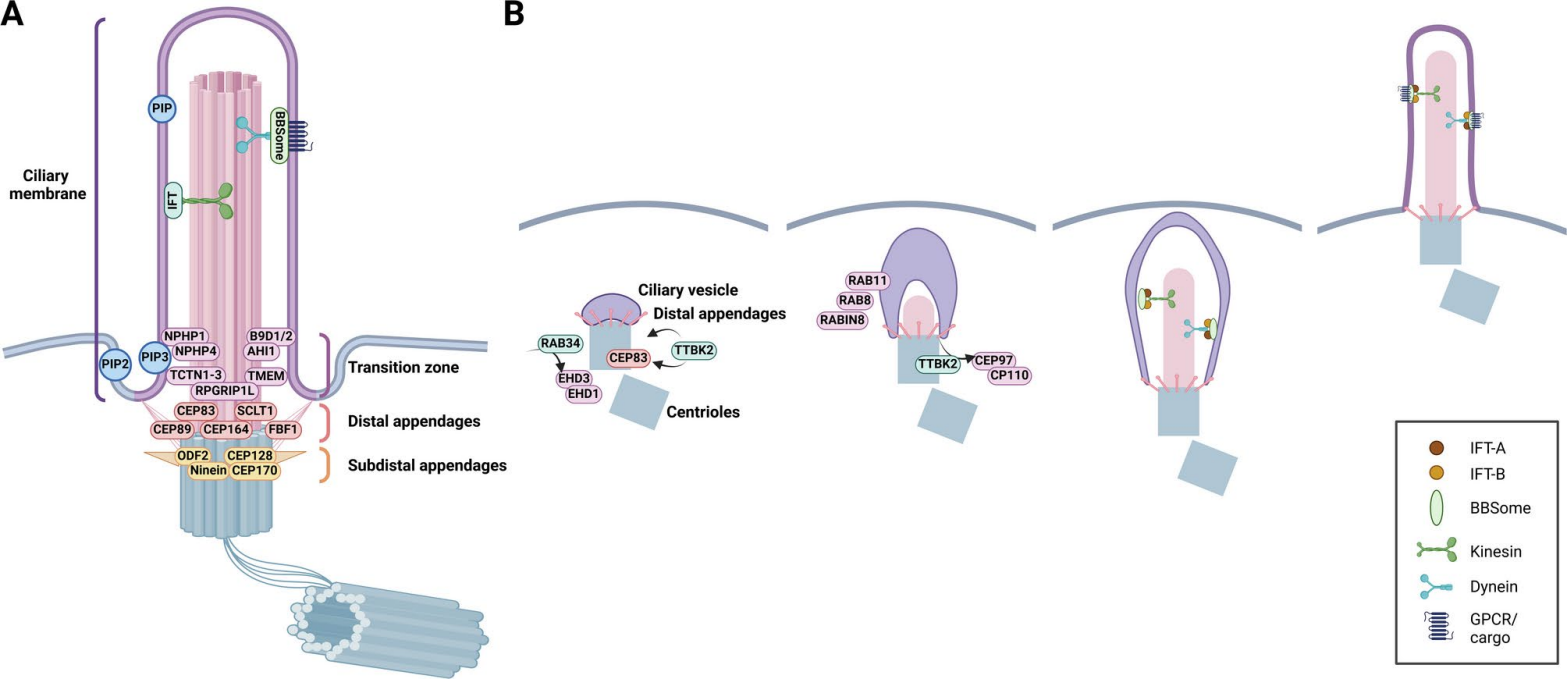
Primary cilium structure and mechanism of intracellular ciliogenesis. **A** Primary cilium originates from the basal body (grey), modified centriole, which possesses two sets of appendages – distal (DAs) and subdistal (SDAs). The central structure is the membrane-enclosed microtubule-based ciliary axoneme (pink). The transition zone (TZ) acts as a diffusion barrier between the basal body and the ciliary axoneme. The ciliary membrane is rich in phosphoinositides (PIP), while phosphatidylinositol 4,5-bisphosphate (PIP2) levels are increased in the periciliary membrane at the cilium base; the phosphatidylinositol (3,4,5)-trisphosphate (PIP3) seems specifically enriched in the membrane surrounding the TZ. Active protein transport is facilitated by intraflagellar transport (IFT) machinery, which works in cooperation with the BBSome – cargo adapter complex. IFT is a bidirectional movement of complexes along the microtubules from the base of the cilium to its tip (anterograde transport) by kinesin motors and from the tip back to the ciliary base (retrograde transport) by dynein motors. **B** Intracellular ciliogenesis begins with the formation of the ciliary vesicle at the distal appendages, a process facilitated by EHD1/EHD3 and RAB34. TTBK2 phosphorylates CEP83 and other DAs components, enabling the ciliary vesicle to dock to the mother centriole. Ciliary vesicle membrane expansion is regulated by the RAB11/RABIN8/RAB8 cascade, driving the development and elongation of the ciliary membrane. Simultaneously, IFT mediates the growth of the axoneme, coordinating its assembly with the extension of the ciliary membrane. This image was created with BioRender.com

The primary cilium consists of the basal body, the transition zone (TZ), and the membrane-enclosed microtubule-based ciliary axoneme (Fig. 1A) [9]. The basal body originates from the older of the two centrioles of the centrosome, termed the mother centriole. Centrioles are barrel-like structures typically assembled according to radial nine-fold symmetry. A major part of their walls is made of nine microtubule triplets arranged in a circular pattern, which contributes to the structural integrity and overall stability of centrioles. The centriole’s most distal part transitions to a geometry reminiscent of an axoneme by containing microtubule doublets instead of triplets and being of smaller diameter than the proximal centriole part [10]. Only a fully matured mother centriole can serve as a basal body, having acquired two sets of appendages [11]. The structural integrity of the centriole distal part is critical for both distal (DAs) and subdistal (SDAs) appendages formation [12, 13].

DAs are present as blades that attach to two adjacent microtubule triplets of the mother centriole wall, forming a structure resembling a nine-fold pinwheel [14]. The DAs blades consist of CEP83/CCDC41, CEP89/CCDC123, SCLT1, and CEP164, which are crucially involved with primary cilia assembly initiation [15, 16] and protein FBF1, involved in regulating ciliary gating [17, 18]. DAs are assembled in a hierarchical manner, with CEP83 being responsible for the recruitment of SCTL1 and CEP89. SCTL1 successively mediates FBF1 and CEP164 recruitment [15]. Mutations in DAs genes (demonstrated for *CEP164*, *CEP83*, and *SCLT1*) typically lead to nephronophthisis, a ciliopathy manifesting in kidneys [5, 6, 19]. Unlike DAs nine-fold symmetry arrangement, SDAs structure does not seem well conserved. Moreover, SDAs are not directly decisive in the regulation of cilium assembly or maintenance; instead, they act as anchorage points for centrosomal microtubules, hence connecting the basal body to the rest of the cell [20]. In turn, they have been implicated in the correct positioning of primary cilia [21], yet functional consequences are not clear. SDAs components include ODF2, Ninein, CEP170, CEP128, and others [20, 22]. Vertebrate cells typically contain only one fully matured mother centriole at a time, allowing for the formation of a single cilium [11]. However, multiciliated cells, including those found in the lung airway epithelium or kidneys, are an exception. During their differentiation, these cells generate up to hundreds of centrioles, which then serve as basal bodies [23].

The transition zone (TZ) delineates the transition between the basal body and the ciliary axoneme [24, 25]. Super-resolution studies have positioned the TZ approximately 100 nm above the DAs [26, 27, 28]. The TZ serves as a diffusion barrier for both membrane and soluble proteins to ensure the unique composition of the intra-ciliary milieu and, consequently, is essential for primary cilia function [25, 29]. Its arrangement and size may vary between different cell types, the common structural feature being the presence of Y-shaped linkers (termed Y-links) that crosslink axonemal microtubule doublets with the ciliary membrane [24, 26]. Many TZ proteins have already been identified [30, 31], most of which are linked to ciliopathies [8], which has then raised even more interest in studying the TZ. Nevertheless, the exact composition of Y-links remains elusive. TZ components cluster into several structurally and functionally distinct protein complexes [32]. One of them is the NPHP complex, which resides in the proximity of axonemal microtubules and consists of proteins such as NPHP1, NPHP4, and RPGRIP1L [25], [32]. Their mutations typically lead to nephronophthisis [8]. A second complex, termed MKS, comprises proteins located in or near the ciliary membrane (e.g., TCTN1-3, several TMEM proteins, AHI1, B9D1/2, etc.) [25], [32]. Corresponding mutations in MKS members are typically involved with Meckel and Joubert syndromes [5], [8]. Both complexes are engaged in several interactions, including mutual ones, interactions with CEP290 (a large protein commonly annotated as another MKS member), and proteins of the so-called Inversin compartment [25]. CEP290 and RPGRIP1L seem to act as the most upstream components in the hierarchy of individual TZ components recruitment [33].

The most common view of axonemal microtubule arrangement in primary cilia is the “9 + 0” configuration, where the central microtubule doublet, typical for the motile cilia with their “9 + 2” arrangement, is missing. While this simplistic model still holds credibility, it is apparent that the spectrum of different configurations is broader than originally anticipated, since the list of reported “exceptions from the rule” is rapidly enlarging. For instance, cilia found in olfactory neurons are immotile, albeit with “9 + 0” configuration [34]. Conversely, beta-cell “primary” cilia, with the typical “9 + 0” configuration, show movement in response to glucose stimulation, likely to facilitate insulin secretion [35]. Moreover, epithelial cell primary cilia seem to follow the “9 + 0” rule only in their proximal parts, while more distal regions of the axoneme exhibit a surprising level of heterogeneity and “disorganization”, with several individual microtubules often ending well before reaching the tip of the cilium [36], [37].

The ciliary membrane is continuous with the plasma membrane, the intermediate zone between them termed the periciliary membrane, which sometimes invaginates to form a ciliary pocket surrounding the proximal part of the axoneme [38], [39]. The ciliary membrane has a unique content of lipids and proteins, critical for correct ciliary functions [29], [40]. Phosphoinositides (PIPs) are perhaps the best example of lipids displaying a precise spatial organization within the cilium membrane. Specifically, the ciliary membrane is rich in PIP, while low in PIP2 and PIP3. Conversely, the plasma membrane has a high content of PIP2 and PIP3. PIP2 levels are increased in the periciliary membrane at the cilium base, while the PIP3 seems specifically enriched in the membrane surrounding the TZ [41]. This particular configuration is maintained by cilia-residing enzymes such as INPP5E, which converts the PIP2 into PIP inside the cilium to maintain the boundary [42], [43].

The soluble content of cilia (recently referred to as cilioplasm [44]) also evinces substantial differences from the composition of cytoplasm. Whereas soluble small molecules easily diffuse between the cilioplasm and cytosol, their concentrations can significantly differ between these two compartments. One such example is calcium, whose steady-state levels can be much higher in the cilioplasm than in the cytoplasm of the same cell [45]. Given that cilium volume is smaller than cell volume by several orders of magnitude, the effects of ciliary ions on cytosol can be safely considered negligible. Conversely, relatively subtle changes (by as little as “a few molecules”) can in fact mean dramatic differences in ion concentration in the context of cilia [46, 47]. For instance, it is estimated that a single molecule of cAMP translates into a 10 nM concentration increase of cAMP inside cilia [44]. This compartmentalization provides a conceptual base for primary cilia as sensitive and efficient signaling organelles [44, 47].

### Primary cilia assembly regulation

Ciliogenesis activation is typical for postmitotic cells, as the cilium is accordingly disassembled prior to the entry of another round of mitosis [9]. Exit from the cell cycle and entry into quiescence is considered the most permissive condition for the assembly of a primary cilium [9]. Commensurate with that, perhaps the most commonly used protocol to facilitate ciliogenesis in mammalian cell culture is based on enriching the G1/G0 cell population via serum starvation [48]. Consequently, primary cilia were considered specific hallmarks of quiescent state [49]. Moreover, certain evidence suggests that primary cilia may even have a checkpoint function - guarding against cell cycle re-entry [50]. Nevertheless, based on data from live cell imaging and in vivo models, primary cilia apparently can be readily found not only in non-cycling but also in proliferating (yet non-mitotic) cells [51], [52]. Actually, vertebrate cells seem capable of assembling primary cilium throughout the cell cycle (except mitosis) [51]. Therefore, the timing of the onset of cilium assembly can be heterogeneous, and factors responsible for such asynchrony within a cell population are currently unknown. The earliest observed event in ciliogenesis, as documented in transmission electron microscopy (TEM) experiments, is the docking of the basal body DAs to the ciliary vesicle (CV) [2, 38] or directly to the apical cell membrane[53]. The former is a feature of the intracellular pathway of cilia assembly, where ciliogenesis already initiates in the cytoplasm, before the mother centriole attachment to the plasma membrane [54], [55]. The latter is common for the extracellular cilia assembly pathway of polarized cells, where ciliogenesis commences following mother centriole docking to the plasma membrane surface. This paper focuses on mechanisms of intracellular ciliogenesis, which is a more broadly used mechanism (Fig. 1B). Specific aspects of the extracellular cilium assembly pathway have previously been thoroughly addressed [56].

The large CV forms from the fusion of smaller vesicles, termed distal appendage vesicles (DAVs), attached to the DAs. Myosin-Va, implicated in the assembly and transport of DAVs, appears to be the earliest vesicle trafficking regulator recruited to the basal body [57]. Large CV formation depends on the coordinated action of two members of the EHD protein family, EHD1 and EHD3 [58]. Recruitment of EHD1/EDH3 to cilia base relies on the activity of small GTPase RAB34, localizing to ciliary sheath transiently forming during the assembly of primary cilia [59, 60]. When these proteins are depleted, DAVs can still dock, but the smaller vesicles fail to fuse, hence halting ciliogenesis [57], [58]. The CV membrane’s subsequent expansion, controlled by the RAB11/RABIN8/RAB8 cascade, leads to the formation and extension of the ciliary membrane, assembled in parallel with the growing axoneme. The small GTPase RAB11 is crucial for delivering vesicles containing RABIN8, a RAB8 guanine nucleotide exchange factor (GEF), to the mother centriole and for regulating RABIN8 activity. Locally enriched RABIN8 ensures the timely activation of small GTPase RAB8, promoting the extension of the ciliary membrane [61], [62], [63, 64]. Noteworthy, very few reports have documented an uncoupling of the growth of the primary cilium membrane and axoneme, suggesting tight cooperation of both processes by yet unclear mechanism [65].

The mother centriole-membrane interaction critically relies on intact DAs [15]. Meticulous TEM analyses showed that DAVs/CV docking is severely compromised when CEP164 [66, 67], CEP83/CCDC41 [68], CEP89/CEP123 [69], and to a lesser extent LRRC45 [70], are depleted. While the roles of SCLT1 and ANKRD26 in DAVs/CV docking have not been directly examined by TEM, SCLT1 is required for the recruitment of RAB34 [60, 71]. One open question concerns the mechanism regulating the DAs-CV interactions. It is plausible that individual DAs components and their domains directly mediate the interactions with CV regulators EHD1/3, Myosin-Va, or RAB34 to support efficient CV formation [71]. Certain aspects of such regulation are under the control of effector protein Tau Tubulin Kinase 2 (TTBK2) [72], recruited to DAs by interaction with CEP164 [73, 74]. The absence of TTBK2 prevents CV-mother centriole interactions, which seems to be partly mediated by its phosphorylation of CEP83 [75]. TTBK2 phosphorylates additional proteins involved in CV- mother centriole interactions (i.e. CEP164, CEP89, RABIN8) [73, 76], however, the functional relevance of this remains unclear.

Cilium growth occurs concurrently with the extension of the microtubule-based axoneme. However, the mechanism that triggers the outgrowth of microtubules from the mother centriole distal end is not entirely clear. A widely accepted hallmark of the onset of ciliogenesis in vertebrate cells is the removal of the centriole distal end proteins CEP97 and CP110 specifically from the mother centriole [77]. CEP97 and CP110 depend on each other for proper localization to the centriole distal end and form a complex that is commonly viewed as a “cap,” possibly sterically hindering the outgrowth of microtubules to form the axoneme [77, 78]. The removal of this “cap” depends on TTBK2 kinase activity [15, 72, 73], [79]. While it is not fully resolved how TTBK2 controls this process, one possibility is that TTBK2 directly acts on the CP110-CEP97 complex to induce its relocalization or destruction. Accordingly, TTBK2 has been shown to phosphorylate CEP97 [80]. Alternatively, TTBK2 might indirectly facilitate the removal of CP110-CEP97 by phosphorylating MPP9 [81].

Although CP110-CEP97 loss from the mother centriole serves as a reliable sign of ciliogenesis initiation in numerous studies, the function of these proteins appears more complex. For instance, depletion of CP110 or CEP97 was reported to cause artificially elongated centrioles [52, 78, 82] or facilitate primary cilia formation in certain cell types [81, 83]. Conversely, some evidence suggests these proteins play a positive role in cilia formation [84, 85, 86]. Therefore, the “cap to prevent microtubule growth” model seems overly simplistic for the emerging context-dependent role of CP110-CEP97.

Axoneme outgrowth is mediated by Intraflagellar Transport (IFT) proteins. IFT is a bidirectional movement of complexes along the axonemal doublet microtubules from the cilium base to its tip (anterograde transport) by kinesin motors (typically kinesin-II) and then from the tip back to the ciliary base (retrograde transport) by dynein 2 motors [87, 88]. The main IFT function is to deliver various cargo (e.g., ciliary components such as tubulin, receptors, etc.) into the cilia and back to promote cilium growth and maintenance. Cargo proteins can interact with IFT complexes directly (e.g. tubulin [89]) or via adaptor proteins [90]. Fully assembled IFT complexes comprise individual IFT proteins forming IFT-A and IFT-B subcomplexes, coupled with respective motor proteins [91]. Cryo-EM work in flagella of *Chlamydomonas* revealed that each microtubule doublet is used as a bidirectional double-track railway - anterograde IFT trains move along B-microtubules, and retrograde trains use A-microtubules for transport [92]. IFT complexes localize to two distinct pools at the ciliary base – one that overlaps with the DAs and the other within the TZ [93]. Their recruitment requires kinase activity of TTBK2 and intact DAs [71, 72], [73], however, exactly which DAs component(s) serve a physical docking platform for the interaction with IFTs is not clear. While IFTs are recruited to DAs even in nonciliated cells [73], their assembly into fully functional “trains“ seems to be completed within TZ, to be subsequently loaded inside the cilium [94, 95].

The transport of molecules inside and outside of cilium is critical for cilium growth, maintenance, and function. The current model posits that TZ together with DAs transformed into so-called transition fibers form a selective barrier between the cilium and the rest of a cell to control the ciliary entrance and exit [5, 20], [25]. Numerous studies have documented the existence of such a gating barrier at the ciliary base, yet the actual mechanism of sorting and transporting selected molecules across the barrier is not fully resolved. According to some evidence, the ciliary entry of various cargo is mediated by IFT activity. In this case, the cargo is simply dragged through the sorting barrier by the activity of IFT motors [5, 29]. In agreement with this model, anterograde IFT trains (likely cargo-loaded) are assembled at TZ, and inactivation of their motors impairs TZ localization of IFT trains and, successively, cilium elongation [5], [90], [95]. This mechanism likely ensures the ciliary entry of tubulin subunits, in cooperation with their free diffusion across the TZ [89, 96]. In addition, membrane-associated proteins and transmembrane receptors seem to enter the cilium independently of IFT motor activity. They rely instead on interactions with IFT-A subcomplex and adaptor protein TULP3 [97, 98]. TULP3 is a PIP2-binding protein, and its ciliary localization is restricted by the PIP2/PIP3 boundary, maintained by INPP5E (which itself is recruited to cilia in a TULP3-dependent manner) [97, 99].

The ciliary exit of many receptors is accordingly facilitated by the BBSome, an octameric cargo adaptor protein complex comprised of BBS1, BBS2, BBS4, BBS5, BBS7, BBS8, BBS9, and BBS18 subunits [64]. Mutations in any of the BBSome subunits lead to ciliopathy termed Barder-Biedl syndrome [100], albeit with various severity across tissues [101]. BBSome formation occurs in a step-wise fashion and is spatially governed by the BBS4 and BBS1 subunits [102, 103]. BBS4 localizes to pericentriolar satellites and recruits here the other subunits to form the pre-BBSome. BBS1 resides at the centrosome and facilitates pre-BBSome translocation and BBSome completion at the ciliary base. Moreover, BBS1 binds RABIN8 and thus serves as a bridge between RAB8/RAB11 dependent vesicular trafficking and the ciliary transport machinery [63, 64].

BBSome seems to be associated with IFT trains in many organisms. As Max Nachury stated, the BBSome may have been easily called IFT-C if not for historical reasons [98]. In fact, BBSome directly regulates IFT machinery movement in *C.elegans* [104]. Whether the analogous function is conserved in vertebrates is currently unclear. Nevertheless, the key function of BBSome, well documented across various model organisms, probably resides in regulating the ciliary exit of membrane receptors (e.g., SSTR3, GPR161, etc.) [98]. According to the current model, BBSome travels together with IFT particles in both anterograde and retrograde directions to retrieve ciliary receptors. Their ciliary exit, namely the passing through the TZ barrier, is subsequently facilitated by interactions with adaptor protein ARL6 (BBS3) [105].

DAs transformed into transition fibers are considered as another module regulating ciliary gating [40]. Such gating function is well expected, considering the intimate association of DAs and the membrane, implied by TEM or super-resolution microscopy studies [2, 17], [28, 38]. However, direct evidence of such function is lacking (and long-awaited), including the details on the interactions between individual DAs proteins and the membrane. One of the limitations is the critical role of several DAs proteins (namely CEP83, CEP164, and SCLT1) and TTBK2 in the initiation of the cilium assembly pathway (see earlier), hence the absence of any of these proteins halts ciliogenesis before any gating mechanism has a chance to step in. There is one exception though - FBF1. Deletion of FBF1 leads to moderate ciliogenesis defect [18, 71], allowing a careful analysis of primary cilia forming independently of FBF1. Indeed, lack of FBF1 in *C. elegans* or human cells causes defective cilia entry of IFT particles [18], together with reduced ciliary localization of SMO, PKD2, and SSTR3 receptors [17, 106]. The exact mechanism(s) that compromise the IFT/receptor recruitment here awaits to be identified.

### Primary cilia disassembly regulation

It is well documented that primary cilium needs to be disassembled prior to the onset of mitosis, most likely to free the mother centriole for centrosome-mediated spindle organization and positioning [9], [107]. Serum-starved NIH3T3, hTERT-RPE1, or IMCD3 cells gradually lose cilia following serum addition [48, 49], [108], and asynchronous NIH3T3 (with ARL13B/Fucci sensor) were shown to disassemble primary cilia at G2/M [51]. An intriguing exception to this otherwise strictly followed rule is the Ptk1 cell line, in which primary cilium is able to persist even during early mitosis [109]. There are at least two modes for disassembling primary cilium. Consistent with the gradual shortening of flagella in *Chlamydomonas* before cell division [110], primary cilia of vertebrate cells can be slowly resorbed by axoneme depolymerization [108]. Moreover, the flagella of many protists can be lost in a process of rapid deciliation, which involves cutting off the ciliary axoneme by the microtubule-severing enzyme Katanin [111]. A similar mode of action also mediates primary cilia disassembly in vertebrate cells. Furthermore, both these modes of primary cilia removal can act separately as well as in combination [108]. While the identification of “switch factors”, controlling which mode of cilia assembly will be used requires additional work, it is becoming clear that the precise timing of cilia disassembly has significant physiological consequences. For instance, primary cilia of neuronal progenitors in developing chick neural tubes undergo timely remodeling of ciliary axoneme, which permits switching between specific branches of the Hedgehog (HH) signaling pathway crucial in neuronal patterning and differentiation [112] (see more on that in the HH signaling pathway chapter).

**Fig. 2:**
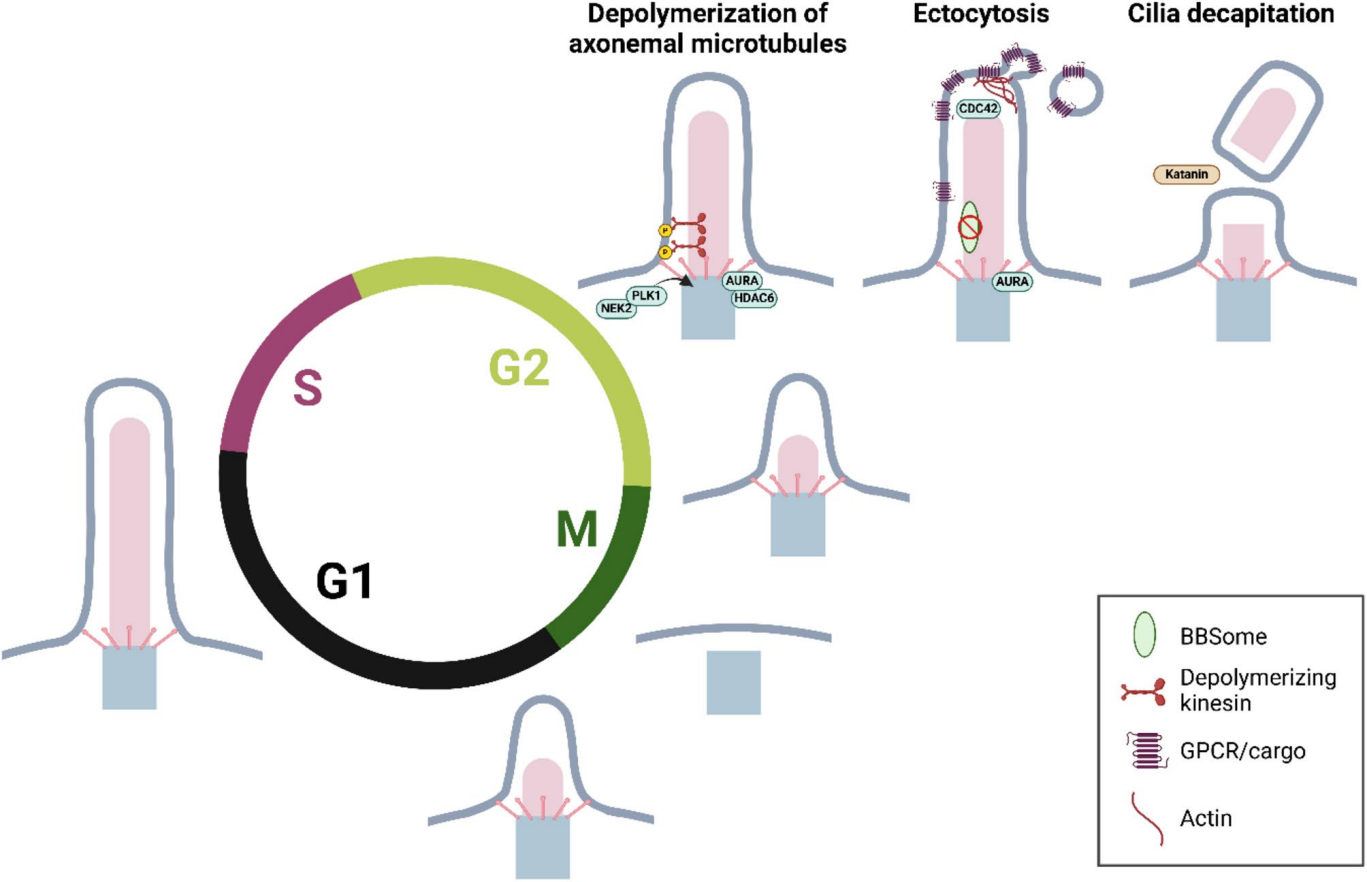
Primary cilium dynamics during cell cycle. Primary cilia are disassembled before mitosis and reassembled after cell division during the early G1 phase. Cilium disassembly occurs through several potentially overlapping mechanisms, including gradual resorption mediated by AURA kinase and HDAC6, CDC42 and actin-dependent ectocytosis, and Katanin-dependent ciliary decapitation. Axoneme (light pink), basal body (grey) with distal appendages (DAs; pink). This image was created with BioRender.com

Gradual cilium disassembly requires destabilization and depolymerization of axonemal microtubules (Fig. 2). The upstream controlling mechanism involves mitotic kinase Aurora A (AURA), whose activity increases during the cell cycle, together with the size of the AURA pool at the base of primary cilia [113]. While the function of AURA upstream of cilia disassembly is well documented and conserved from *Chlamydomonas* to vertebrates [113, 114], the underlying mechanism is less clear. Available evidence from cell culture-based experiments suggests that a subset of histone deacetylates including HDAC6 or HDAC2, able to deacetylate tubulin, acts downstream of AURA to mediate the destabilization of axonemal microtubules [113, 115]. However, genetic ablation of HDAC6 or other HDACs does not lead to major ciliary phenotypes in vivo, indicating the involvement of compensatory actions and/or cell type specificity of the outlined mechanism [116, 117]. Still, considering both AURA and HDAC6 are enriched at the cilia base (with currently no evidence of their localization in the ciliary axoneme), one of the outstanding questions is how the signal from AURA/HDAC6 passes to the axonemal microtubules. A possible (though highly speculative) explanation may relate to the fact that HDAC6 prefers tubulin dimers over polymerized microtubules [118]. In such a scenario, HDAC6 would primarily act on tubulin subunits transported through the barrier at the cilia base.

Disassembly of the primary cilium before mitosis is further controlled by several members of the kinesin 13 family (e.g., KIF2A, and KIF24). Unlike conventional kinesin motor proteins, kinesin 13 proteins do not “walk” along microtubules but possess the unique activity of ATP-dependent microtubule depolymerization to promote resorption of ciliary axoneme [119]. KIF2A is enriched at the base of primary cilia in the close vicinity of DAs/SDAs, and can also be found in the axoneme [79], [120]. The ability of kinesin 13 proteins to depolymerize microtubules is tightly controlled by upstream kinases. Phosphorylation by mitotic kinases such as PLK1 promotes its activity [120], while phosphorylation by TTBK2 counteracts KIF2A recruitment to the ciliary base and hampers its microtubule-depolymerizing activity [79, 121]. Similarly, the activity of axoneme-depolymerizing kinesin KIF24 is promoted by phosphorylation by NEK2 [122].

Another phenomenon, termed ectocytosis or cilia decapitation, has been recently linked to the regulation of primary cilia dynamics, eventually leading to their disassembly [123], [124] (Fig. 2). Here, events like the defective exit of ciliary receptors owing to impaired BBSome function [124], [125], or mitogen-induced INPP5E removal from primary cilia [123], trigger remodeling of ciliary actin and, accordingly, myosin-mediated abscission of the ciliary membrane and its release from the tip of cilia in a form of a small vesicle. Consequently, this may cause gradual resorption of the whole ciliary axoneme. Intriguingly, INPP5E removal from cilia and, successively, the shortening of ciliary axoneme requires AURA activity [123, 126], providing another means of how AURA activation contributes to primary cilia disassembly. Here, the shortening of primary cilia caused by BBSome deficiency can be reversed through both AURA and the intraciliary RHO GTPase CDC42 [125, 127], suggesting a close interplay between axoneme resorption and ciliary membrane turnover.

## Crosstalk of cilia assembly and disassembly pathways with other regulatory mechanisms

The previous paragraphs have illustrated that primary cilia are highly dynamic organelles. In fact, as little as 5 min of halted IFT is enough to detect changes in primary cilia composition. Therefore, perhaps unexpected heterogeneity and asynchrony related to the initiation of their assembly and disassembly, respectively [51, 108], possibly reinforce a notion that having/not having a cilium is a result of counteracting interactions of pro-assembly and pro-resorption pathways. In this section, we plan to discuss some of these interactions thoroughly, including the relationship between cilia formation/resorption and cell cycle, the crosstalk between individual regulatory modules in cilium biology, along with the possible dual (non-ciliary) role of primary cilia regulators.

### Cell cycle and primary cilia

As previously noted, primary cilia were originally viewed as hallmarks of quiescence, perhaps even having a checkpoint function. According to this model, primary cilium had to be resorbed before cell cycle re-entry and, conversely, a block of primary cilia disassembly led to cell cycle arrest [50]. While this model has gained some attention, especially when considering the possibility of halting tumor growth by inducing ciliogenesis in cancer cells, it remains controversial, with conflicting data in the literature. Actually, this raises a conundrum: How can organelle, resorbed prior to cell cycle re-entry, still convey proliferative signals (e.g. by HH ligands) important for G1-S transition? [9] Specifically, scaffolding protein trichoplein (TCHP) has been proposed to promote G1-S transition in ciliated cells by activating AURA and then dissembling cilia [128]. This model, however, has been challenged in a report indicating that TCHP regulates cell cycle progression independently of cilia status [129]. With the wide adaptation of live cell imaging microscopy, many reports have subsequently demonstrated the presence of primary cilia in cycling cells [9], [51, 130]. Still, considering the heterogeneity in the timing of primary cilia assembly/disassembly, the possibility of some feedback mechanism between primary cilia and cell cycle, eventually leading to cell cycle alterations between ciliated and non-ciliated cells cannot be completely dismissed.

**Fig. 3:**
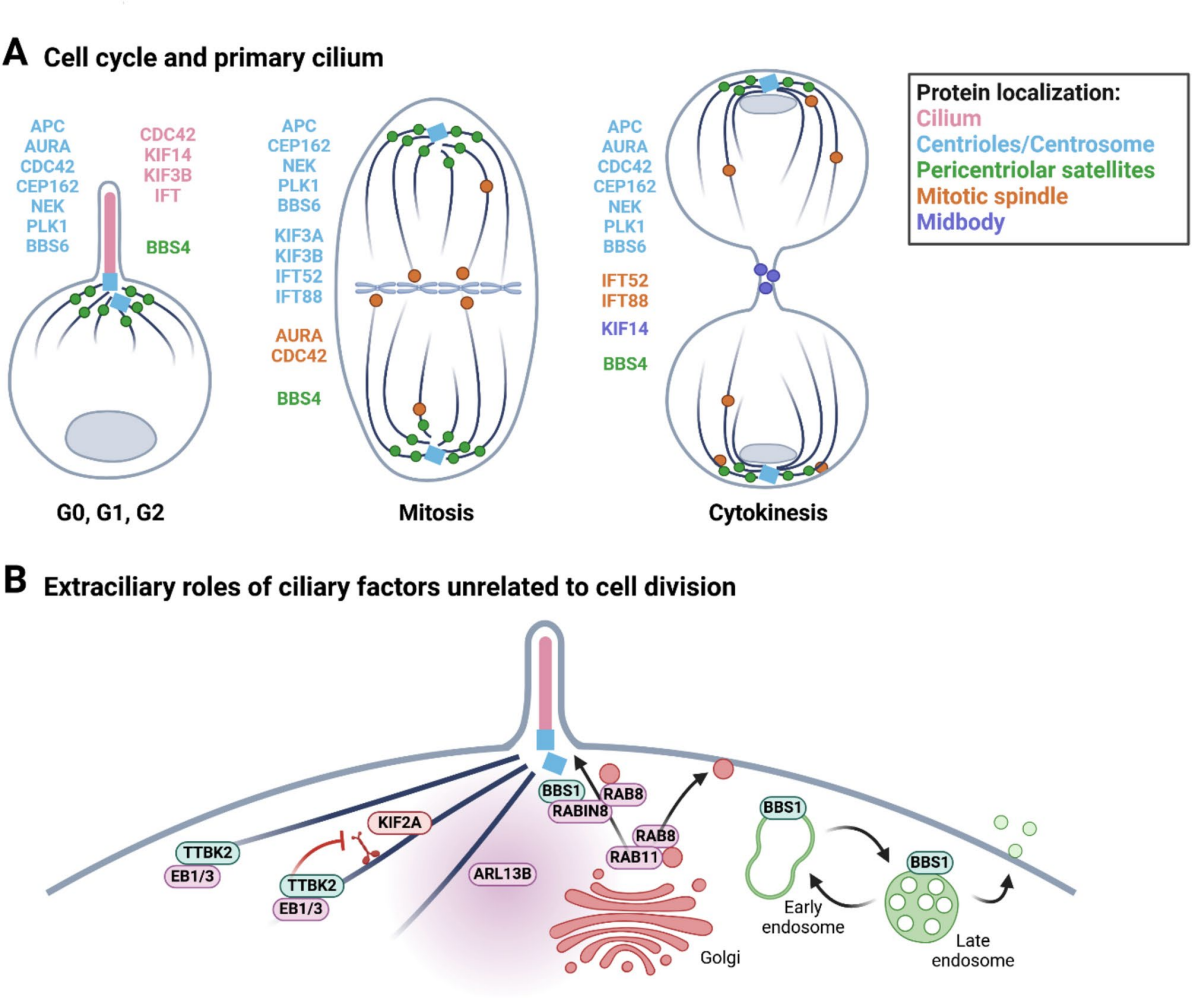
Dual roles of cilia regulators in cell cycle and intracellular organization. **A** Established mitotic regulators such as AURA, NEKs, APC, CDC42, and PLK1 also play critical roles in regulating cilium disassembly. Common ciliary factors such as IFTs (e.g. IFT52 and IFT88), KIF3A/B, KIF14, CEP162, BBS4, and BBS6 also facilitate the formation of the mitotic spindle, chromosome alignment, and the progression of cytokinesis. **B** The factors involved in ciliogenesis and cilia function are vital to intracellular organization. TTBK2 binds to EB1/3 and promotes microtubule growth by suppressing depolymerizing activity of the KIF2A kinesin. ARL13B regulates the non-canonical Hedgehog (HH) pathway and is key in controlling actin organization. The RAB11/RABIN8/RAB8 cascade controls polarized vesicular trafficking to the plasma and ciliary membrane, where the latter is mediated by the BBS1-RABIN8 module. BBS1 is also involved in the endomembrane vesicular trafficking between the early and late endosomes. This image was created with BioRender.com

While direct evidence for the checkpoint role of primary cilia is currently lacking, the intimate relationship between primary cilia and the cell cycle is well documented (Fig. 3A). One aspect of this interconnection is the effect of cell cycle regulators on primary cilia dynamics. The key players here are proteins well known for their prominent roles in mitosis. They are typically associated with the centrosome/ciliary base throughout the cell cycle, starting with very low abundance at G1 and peaking their levels/activity in the G2 or M phase. These include, for instance, AURA (see above) and NEK kinases [131], as well as an anaphase-promoting factor (APC) [132]. Similarly to AURA, the activities of NEK kinases have been shown to promote cilia shortening/disassembly, while their depletion leads to ciliary axoneme elongation. These effects were observed in several models, including *Chlamydomonas* and *Tetrahymena*, as well as in mammalian cells, suggesting the role of NEKs is well conserved [131]. Mechanisms underlying the effects of NEK kinase activity include destabilization of microtubules [131] or, in the case of NEK2, disassembly of DAs [133]. The ciliary role of APC seems to be mediated by targeting NEK1 for degradation [132]. Interestingly, some mitotic regulators can act as ciliogenesis-promoting factors, e.g., by facilitating centriole maturation. One such example is PLK1, whose ectopic activation in interphase erases differences between mother and daughter centrioles (the daughter prematurely assembles DAs), and then, allows the formation of two primary cilia per cell [134]. Considering the orderly nature of the cell cycle, heterogeneity in the timing of primary cilia assembly/disassembly seen in several time-lapse experiments is somewhat unexpected. It implies that rather than providing an instructive signal coupled to a specific checkpoint, the cell cycle creates a permissive window for cilia assembly, fine-tuned by (potentially stochastic) fluctuations in the activities of key regulators.

Perhaps the best-described functional connection between cilia and cell cycle is the extensive proliferation leading to cyst formation in kidneys – a hallmark feature of several ciliopathies[6]. In fact, kidney cyst formation, caused by mutation of cilia residing receptor polycystin 1/2 (PKD1/2) or DAs protein CEP164, can be mitigated by inhibition of cyclin-dependent kinases[19], [135]. Aberrant proliferation related to PKD1/2 mutations seems to be caused by deregulated Ca^2+^ signaling and cAMP production[136]. Another prominent driver of cell proliferation in kidneys is the Hedgehog (HH) signaling pathway. The causative relationship between cilia defects, deregulated HH signaling, and kidney cyst formation has been indicated for mutation of the IFT-A component, IFT139, that leads to bulbous cilia and an overactivated HH pathway[137], [138].

 Another aspect of primary cilia–cell cycle connection is the role of several cilia components in cell cycle regulation, particularly during mitosis and cytokinesis. These effects appear to represent additional functions of these proteins, independent of primary cilia. Both mitosis and cytokinesis rely on the action of microtubules and associated proteins associated. Kinesins and other microtubule-binding proteins therefore represent a group where such cilia-unrelated function is anticipated[139]. KIF3A and KIF3B constitute the kinesin-II heterodimer, which, besides participating in the anterograde IFT in cilia, represents one of the most ubiquitously expressed kinesins, implicated in many forms of intracellular transport [140]. Furthermore, KIF3A/3B is associated with mitotic spindle in both *Chlamydomonas* and vertebrate cells, and expression of a mutated form of KIF3B leads to mitotic abnormalities and aneuploidy [141]. A different example is another microtubule-plus end motor, KIF14, originally identified as cytokinesis-regulating kinesin associated with the midbody [142, 143]. However, during interphase, KIF14 localizes to the axoneme of primary cilia to regulate cilia growth, IFT, and competence to convey HH pathway activation [144, 145]. The association between mitotic apparatus and cilia regulators extends to IFT and BBS proteins (see Table 1 for a detailed overview). This is perhaps the best documented for IFT88 (member of IFT-B complex). In dividing cells, IFT88 localizes to the mitotic spindle, where it takes part in a dynein1-driven complex to ensure the correct formation of astral microtubule arrays and spindle orientation. Consequently, IFT88 depletion leads to mitotic defects in cell culture cell lines, mice, and zebrafish [146]. Moreover, IFT88 has been implicated in the re-localization of AURB during cytokinesis [147], and, together with another IFT-B component, IFT52, in the clustering of supernumerary centrosomes in cancer cells [148]. Also, IFT proteins from both IFT-B and IFT-A subcomplex re-localize from basal bodies/cilia to cleavage furrow in dividing *Chlamydomonas* [149]. Interestingly, IFT-A and KIF3A have another way to affect the cell cycle independently of cilia – via regulation of the WNT/β-catenin pathway [150], [151]. Our WNT signaling chapter has further details. Other important primary cilia biology regulators with ties to proliferation control are BBS proteins. One of the initial BBS studies, which linked BBS4 to primary cilia and centriolar satellites, actually reported that BBS4 depletion leads to cell cycle arrest and prominent cytokinesis defects [152]. This proposed mechanism involves BBS4-PCM1 interaction in centriolar satellites and, accordingly, defective anchoring of microtubules in BBS4-depleted cells. Similarly, chaperonin BBS6 exhibits prominent localization to centrosome and centriolar satellites, and its depletion induces cytokinesis defects and the appearance of multinucleated cells [153]. One more example of a ciliary component with a cilia-unrelated role during cell division is CEP162, a protein with microtubule-binding ability residing at the centriole distal end to promote and restrict the TZ formation [154]. Additionally, CEP162 localizes to spindle microtubules and centrosomes in dividing cells, and its loss leads to abnormal mitosis and chromosome segregation defects [154], [155].

**Table 1:** Cilia regulators with dual roles

Protein(s)	Centrosome/cilia -related function	Extraciliary functions - cell cycle	Extraciliary functions- other
**AURA**	cilia disassembly [113, 114]	mitotic entry, spindle assembly, chromosome alignment [157]	
**HDAC6**	cilia disassembly [113, 115]		microtubule deacetylation [116, 117]
**CDC42**	cilia shortening, actin-dependent ectocytosis [125]	chromosome alignment [157]	regulator of cell polarity and actin-based morphogenesis [158]
**NEK kinases**	cilia shortening/disassembly [131]	destabilization of MTs [131] disassembly of DAs [133]	
**APC**	stability of axonemal microtubules through targeting NEK1 [132]	anaphase promoting complex [157]	
**PLK1**	centriole maturation [134]	mitosis progression [157]	
**KIF3A** **KIF3B**	anterograde IFT [140]	intracellular transport [140] mutations in KIF3B - mitotic abnormalities and aneuploidy [141]	intracellular transport [140]
**KIF14**	cilia growth and trafficking, HH activation [144]	localization to midbody, cytokinesis progression [142, 143]	
**IFT88**	ciliogenesis, IFT transport [87]	spindle orientation, AURB positioning during cytokinesis [147]	clustering of centrosomes in cancer cells [148]
**BBS4**	BBSome-dependent trafficking [152]	cell cycle progression and cytokinesis [152]	pre-BBSome assembly [102] exosome release [235]
**BBS1**	BBSome-dependent trafficking, ciliary gating by supporting TZ structure [102]		endomembrane trafficking [162, 163]
**BBS6**	BBSome assembly [153]	progression of cytokinesis [153]	CCT/TRiC-chaperonin complex [153] exosome release [235]
**CEP162**	TZ formation [154]	chromosome segregation during mitosis [155]	
**TTBK2**	primary cilia assembly [73–77, 80, 173, 174]		MTs dynamics and cell migration [121]
**RAB11** **RAB8** **RABIN8**	polarized trafficking of ciliary vesicles, outgrowth of ciliary membrane and ciliogenesis [61, 62, 64]		endosomal recycling, polarized transport of Golgi-derived vesicles [63, 159, 160]
**ARL13B**	ciliary membrane composition; ciliary targeting of INPP5E and HH pathway components [165–167]		non-canonical HH signaling essential for the axon guidance [168, 169]

Summarily, available evidence suggests that cells are efficiently re-purposing their regulatory elements, leading to a shared toolkit of regulators of cilia and cell cycle. Proteins commonly viewed as mitotic regulators (AURA, NEKs, APC) mediate the cilia disassembly to ensure cilia do not intervene with spindle function during cell division. Conversely, proteins involved in cilia biogenesis (IFT, BBS, KIF3A/3B, KIF14, etc.) often take over new tasks during mitosis or cytokinesis, as there is no cilium to care about at the moment. This opens an interesting possibility that some of the many pathologies found across different “ciliopathies” may in fact stem from non-ciliary functions of cilia regulators [156].

### Extraciliary roles of ciliary factors

TTBK2 represents an example of a critical regulator of primary cilia assembly, with an additional role outside of cilia, not directly linked to cell division (Fig. 3B; Table 1). Outside of the basal body, TTBK2 interacts with microtubule plus-end tracking proteins (+ TIPs) such as EB1/3 to localize to cytoskeletal microtubules and regulate their dynamics and successive cell migration [121]. Interestingly, at least some activities of TTBK2, both inside and outside of primary cilia, rely on a common mechanism - phosphorylation and subsequent inactivation of microtubule-depolymerizing kinesin KIF2A [79], [121].

RAB11/RABIN8/RAB8 pathway activity is not restricted to primary cilia and is also involved in several aspects of vesicular trafficking in the cytosol, including endosomes recycling or transport of Golgi-derived vesicles to the cell membrane [63], [159], [160]. The BBS1 subunit of the BBSome interacts with RABIN8, linking the BBSome to the vesicular trafficking of ciliary proteins to the primary cilium base [64], [161]. BBS1 has also been detected on early and late endosomes in HEK293 cells [162], suggesting that BBSome is more broadly involved in the endomembrane system beyond its function in ciliary transport [163].

A surprising non-ciliary function has also been identified for ARL13B, which is perhaps the most commonly used marker to visualize primary cilia in microscopy-based experiments, and its mutations cause Joubert syndrome [48], [164]. ARL13B is a membrane-associated small GTPase of the ARF/ARL family, important for correct ciliary targeting and localization of INPP5E and several HH pathway components [165], [166], [167]. Recent work from the Caspary lab described ARL13B^V358A^ mutant, which is unable to localize to primary cilia, and the cilia are shorter and show diminished INPP5E levels. Notably, ARL13B^V358A^ still seems fully capable of rescuing major HH phenotypes related to ARL13b loss in mice [168], [169]. While ARL13B^V358A^ is missing in primary cilia, it could be identified in axons and growth cones of commissural neurons, where it probably mediates non-canonical HH signaling relevant for axon guidance [169]. Considering the prominent role of ARL13B in disease and the well-supported model of HH pathway dependence on primary cilia in vertebrates, it would be very interesting to test the ability of ARL13B^V358A^ to mediate HH signaling in a model with ablated ciliogenesis.

### Interactions between individual modules of primary cilia

**Fig. 4:**
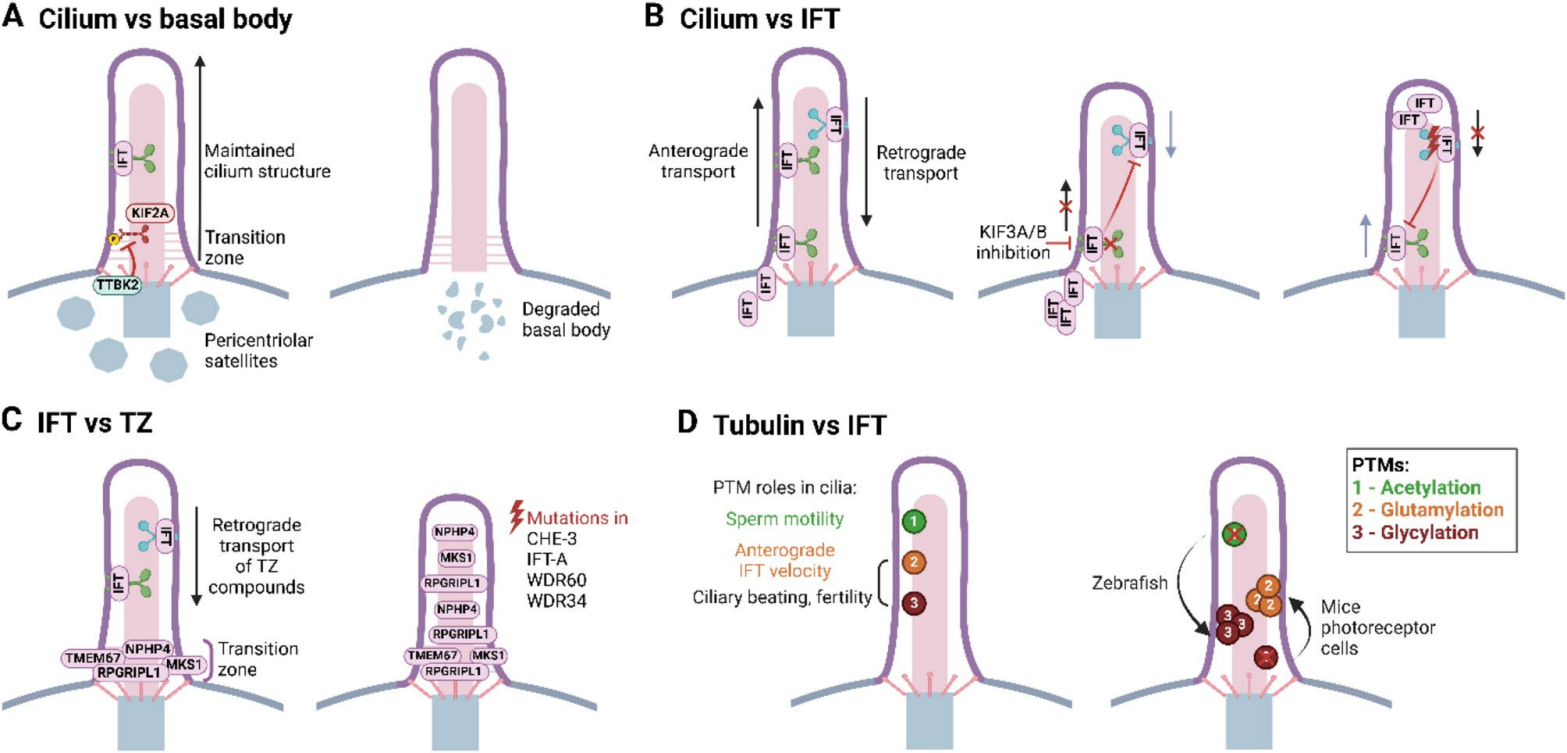
Interactions between individual modules of primary cilia. **A** The basal body and pericentriolar satellites are crucial for the growth and maintenance of the ciliary axoneme. TTBK2 at the basal body promotes cilium growth by suppressing the depolymerizing kinesin KIF2A. In *C. elegans*, the basal body is degraded once the cilium is assembled. **B** Anterograde and retrograde IFT display functional crosstalk in cilium length control. Inhibition of the heteromeric KIF3A/3B kinesin II disrupts anterograde IFT and indirectly halts retrograde IFT, causing IFT particles to accumulate at the ciliary base. Inhibition of the dynein motor by ciliobrevin D or mutation in IFT-A complexes indirectly abrogates anterograde IFT. **C** Retrograde IFT is crucial for correct TZ establishment. Mutations in components of retrograde IFT lead to the mislocalization of TZ components inside the cilium, and the shortening of cilia. **D** Posttranslational modifications (PTMs) of axonemal tubulin are crucial for cilia function. Axoneme acetylation, glutamylation, and glycylation are involved in cilia beating and sperm motility, likely modulating the velocity of the anterograde IFT. PTMs can also compensate for each other; axoneme hyper-glycylation can compensate for axoneme deacetylation in zebrafish cilia, while axoneme hyper-glutamylation can compensate for axoneme deglycylation in murine photoreceptors. This image was created with BioRender.com

In the following section, we will discuss how individual cilia compartments affect each other to control the dynamics of the fully assembled organelle. We begin with the basal body, which is typically viewed as an inseparable part of cilium, and, via the DAs-associated activities previously noted, is critically involved in primary cilia assembly initiation (Figs. 1A and 4A). In the primary cilia of sensory neurons of the nematode *C.elegans*, the basal body is degraded following cilium assembly[170]. In fact, centrioles are lost in most cells in *C. elegans*, coinciding with the cessation of proliferation. Even though the basal body is lost, transition fiber-like structures still connect the proximal axoneme end with the membrane [170], indicating a function of this compartment exceeding ciliogenesis initiation. Indeed, emerging evidence from mice and cell culture cell lines has established DAs kinase TTBK2 capacity in primary cilia maintenance. TTBK2 activity ablation after cilia assembly effectuates their shortening, perturbed localization of IFT proteins and HH pathways components, and axonemal microtubules glutamylation reduction [171, 172]. Proposed mechanisms downstream from TTBK2 involve KIF2A inhibitory effects [79] and centriolar satellite remodeling [172].

Dynamic interactions are also typical for the IFT. As noted earlier, disruption of IFT-dynein or IFT‐A complexes results in short, bulged primary cilia due to the accumulation of IFT proteins and their cargo. Conversely, mutations in IFT‐B components typically result in complete axoneme assembly failure [87]. Interestingly, while the general phenotypes of anterograde and retrograde defects, respectively, are well distinguishable, emerging evidence also suggests “crosstalk” between anterograde and retrograde transport (Fig. 4B). The putative sites for such interactions are the ciliary base and tip, respectively, where IFT trains rearrange to switch motors [87]. Acute inhibition of heteromeric KIF3A/3B kinesin II, for instance, completely halts both anterograde and retrograde transport in primary cilia in NIH3T3 cells within minutes [173]. However, dynein motor inhibition by ciliobrevin D halts anterograde IFT trains within 30 min of stalled retrograde transport [174]. Furthermore, CHE‐3, the *C. elegans* orthologue of the retrograde IFT‐dynein‐2 motor DYNC2H1 demonstrably affects both retrograde and anterograde transport [175]. Similar observations were reported in WDR60 and WDR34 dynein-2 mutants using mammalian cell lines [176]. One possible explanation for these phenotypes is the roadblock/traffic jam model, where stalled IFT trains impair “opposing” IFT train movement indirectly, by sterically limiting the “free road” available. Considering IFT-A and IFT-B trains show direction-specific usage of the two microtubules in a doublet to avoid collisions, at least in *Chlamydomonas* [92], reported reciprocal interactions between anterograde and retrograde transport machinery may indicate a more complex relationship. The modeling of IFT motor capacity to walk through crowded environments or pass obstacles warrants future investigation.

We have previously discussed the IFT trains being assembled at TZ and noted that IFT-A proteins participate in the import of membrane receptors through the TZ gate. Interestingly, IFT-TZ interactions seem to be even more complex with available evidence further implying IFT involvement in TZ assembly/maintenance regulation (Fig. 4C). The initial evidence of IFT involvement in TZ formation comes from *Trypanosoma*, where IFT mutations precipitate TZ shortening [177]. The requirement of functional IFT for correct TZ function is conserved in metazoan. Ablation of WDR60 or WDR34 in hTERT-RPE1 cells affects the expansion of RPGRIPL1 from the TZ inside the cilium and reduces the TMEM67 pool in the TZ [176]. Similarly, ectopic localization (expansion from the TZ into the proximal axoneme) of several TZ components is connected to the ablation of dynein motor CHE-3 in *C. elegans* [175]. Furthermore, such ectopic localization of several TZ components has also been established for several IFT-A mutants in *C. elegans* [170]. The functional consequence of IFT-TZ interactions is the prominent worsening of gating function in worms with ablated IFT and MKS over the single mutants [178]. One possible explanation of IFT effects on TZ is that TZ proteins begin their assembly in the most proximal region of the axoneme following ciliogenesis onset, yet a portion of these proteins can become mislocalized to distal ciliary regions and potentially create ectopic axoneme–membrane connections. The IFT retrograde machinery purpose may therefore be to assist in the retrieval of mis-localized TZ proteins [175], [178]. Interestingly, the antithesis of IFT-TZ interaction is that disrupting the TZ NPHP module restores the ciliary exit of “underpowered” retrograde trains in WDR-60 mutant, which otherwise accumulate inside the cilium [179]. The model of mutual regulation of IFT-TZ has support in association (though rare) of eye and kidney phenotypes, typical for TZ defects, with IFT mutations [8], [180].

New paradigms are emerging on possible mutual interactions of the ciliary axoneme and IFT motor proteins. Modulation of microtubule tracks in the ciliary axoneme, which fine-tunes the motor proteins walking on them, could be explained by the so-called “tubulin code”. The overall concept behind this term is that molecular patterns generated by combinations of tubulin isotypes and their posttranslational modifications (PTMs) such as acetylation, (poly)glutamylation, (poly)glycylation, detyrosination, etc., control the functions of microtubules [181], [182]. Almost all of the amino acid and length variation between different tubulin isotypes is confined to their unstructured C-terminal tails (CCTs). Consequently, the presence and absence of the CCT, respectively, and the type of its modification have substantial effects on the motility of several kinesin motors (including kinesin II) on purified microtubules in vitro [181]. Ciliary axonemes of both primary and motile cilia are rich in several tubulin PTMs [181], [184], which has been commonly associated (although not yet completely proved) with their considerably higher stability over the cytosolic microtubules. Thus, “guilt by association” evidence suggests that tubulin PTMs play a role in cilia biology (Fig. 4D). Indeed, several of these PTMs show non-uniform, pattern-like distribution in the axoneme [184]. The function of tubulin PTMs is reasonably well-documented for the motile cilia, where the glycylation and glutamylation are particularly enriched in the B microtubule of the doublet [184]. However, the role of tubulin modifications in primary cilia biology is less clear [181]. Polyglycylation of tubulin CCT is almost exclusively found in ciliary axonemes over cytosolic microtubules [181], and is essential for ciliogenesis, at least in protists. *Tetrahymena* strains with defective polyglycylation (where tubulin encoding genes were replaced with variants lacking corresponding glycylation sites) have profound defects in axoneme structure and exhibit impaired movement of flagella [182]. The situation seems to be different in mammals. Elimination of enzymes responsible for tubulin glycylation (TTLL3 and TTLL8) results in the absence of glycylated tubulin in cilia, albeit without a penetrant cilia-related phenotype (mice are viable and of normal morphology). Glycylation, therefore, does not seem to be critically involved in the assembly or maintenance of primary cilia. Instead, the defects in TTLL3/TTLL8 double knockout are restricted to the sperm flagella, inducing altered beating dynamics and then reduced fertility [185]. Similarly, aberrant (poly)glutamylation is linked to defective cilia beating in both *Tetrahymena* and mammals (where it often manifests in fertility and respiratory defects) [181], [186]. Significantly, axoneme glutamylation has been implicated in the regulation of the velocity of several ciliary motors and the release of cilia-derived extracellular vesicles in *C.elegans* [187]. Moreover, chemically-induced cilia de-glutamylation led to a slower anterograde movement of IFT88 containing trains in primary cilia of NIH3T3 cells [188]. If confirmed in other model systems, the glutamylation-IFT link could provide long-anticipated, yet currently lacking evidence of the functional impact of glutamylation/tubulin code on primary cilia biology. The purpose of acetylation in cilia is currently unclear, despite the fact that tubulin acetylation was the first described tubulin PTM [181]. Enzyme αTAT1 has been identified as the major regulator of tubulin acetylation, its ablation causing a loss of this PTM in mice, however excepting moderate fertility defects related to sperm motility, the mice are viable and develop normally [189]. Surprisingly, the cytosolic microtubules exhibited higher stability in the absence of αTAT1 [189]. We use this observation as a proxy to present another emerging phenomenon in tubulin PTMs regulation – compensatory mechanisms at the level of “writers”, “erasers”, or even between individual types of tubulin PTMs, which likely contribute to the lack of gross cilia phenotypes in various models. Loss of tubulin glycylation in mouse photoreceptors is accompanied by an increased glutamylation, perhaps due to competition of the corresponding enzymes for the same modification sites in the CTT [181]. Mutation of three enzymes responsible for tubulin deacetylation (SIRT2, HDAC6, and HDAC10) brings about the anticipated reduction of tubulin acetylation in cytosolic microtubules, yet concurrently decreased acetylation and increased glycylation of ciliary axonemes in zebrafish, and, furthermore, without a notable impact on ciliary function [117]. Clearly, resolving the nature of compensatory mechanisms is a crucial step in assessing the functional impact of tubulin PTMs on primary cilia biology. Since we have now discussed means in which the axoneme may affect walking motor proteins, it is worthwhile noting a rather hypothetical, yet very intriguing possibility that motor protein may communicate back to the cilium MT tracks. This new concept, currently demonstrated on prototypical cytosolic kinesins, kinesin 1 and 4, suggests that motors can not only induce conformational changes in tubulin owing to their walk, but the stepping may actually induce damage in the microtubule, which can be subsequently repaired by incorporation of free tubulins in the damage sites [190].

Substantively, three areas await further consideration: Whether this phenomenon additionally applies to ciliary kinesins, its potential impact for IFT, and whether the cilium developed efficient countermeasures to compensate/eliminate kinesin-induced changes in microtubule tracks.

## Adaptive changes in primary cilium structure and composition to cell signaling pathways

Cilia are typically categorized into primary cilia (which are immotile and serve to sense and transduce signals), and motile cilia (which move extracellular fluids). However, increasing research reveals that both primary and motile cilia can modulate key developmental signaling pathways, such as HH, and WNT [191], [192]. Likewise, certain primary cilia can exhibit limited or specialized motility under specific conditions. As previously noted, pancreatic beta cells possess so-called hybrid cilia, which move to sense glucose levels and promote insulin production in the pancreas [35]. A temporally regulated change in primary cilia motility has been observed during the perinatal period of the choroid plexus development [193]. This transient motility shift was accompanied by changes in gene expression, suggesting a unique role in choroid plexus formation at this stage [193]. During tissue and organ development, cilia can change their identity and also undergo retraction and reassembly [127], [194], [195]. Moreover, emerging evidence suggests that cilia physically interact with neuronal axons via synapse-like connections [196]. These and other findings emphasize the need to view cilia as dynamic organelles with complex, context-specific adaptations in homeostasis, development, and regeneration. Recently, cilium’s involvement in developmental signaling pathway transduction was reviewed in great detail [3], [5]. At this juncture, we will explore how specific signals shape cilium composition and structure and accordingly, how cilia are associated with key signaling pathway regulations, crucial for cell differentiation and tissue morphogenesis.

### Hedgehog signaling

**Fig. 5:**
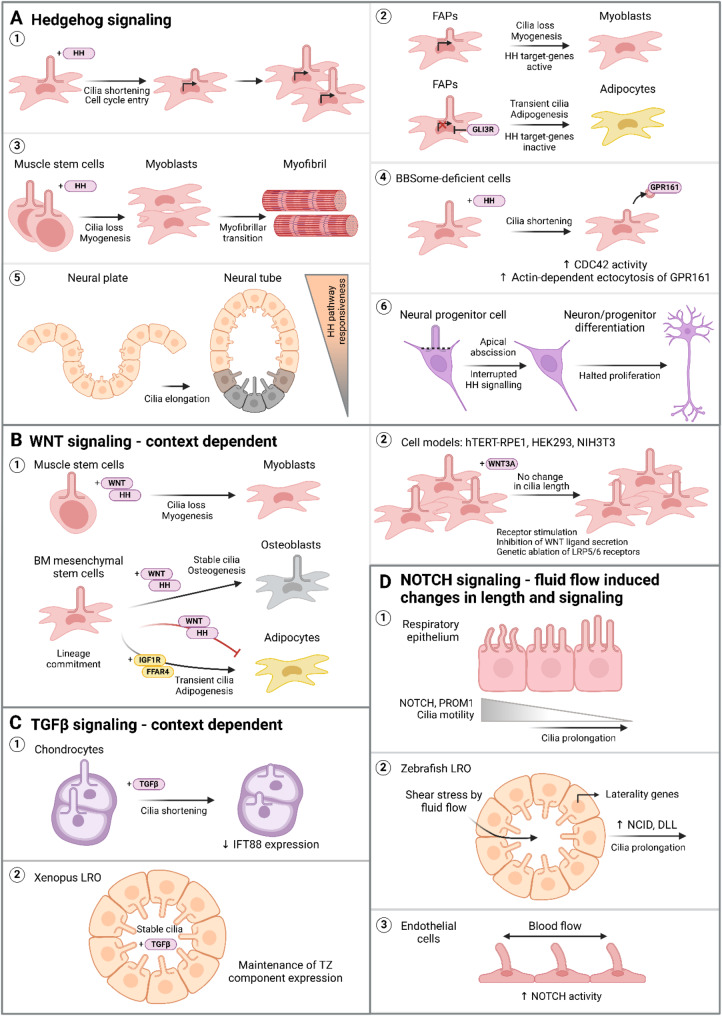
Adaptive changes to cilia dynamics during cell signaling. **A** Activation of HH signaling triggers cilia shortening and promotes cell cycle entry, passing on this effect to the subsequent progeny (1). In fibro/adipogenic progenitors (FAPs), the length of cilia modulates muscle regeneration. Short or absent cilia on FAPs block adipogenesis and promote myogenesis, while long cilia favor adipogenesis (2). Similarly, HH signaling induces myoblast differentiation from muscle stem cells, where myoblasts lose cilia to form myofibrils (3). In BBSome deficiency, HH signaling triggers CDC42/actin-dependent ectocytosis of ciliary GPR161, leading to subsequent cilia shortening (4). After neural tube closure, HH-producing cells elongate cilia to attenuate their responsiveness to the HH pathway (5). During differentiation of neural progenitors, apical abscission of the ciliary axoneme from the basal body leads to a temporary cilia dysfunction. **B** The effects of WNT signaling on primary cilia dynamics are context-dependent. During tissue differentiation, WNT functions together with HH to either stimulate cilia disassembly in myoblasts or sustain cilia dependent signaling in bone marrow mesenchymal stem cells (BM MSCs) during osteogenesis (1). In MSCs, WNT and HH act inhibitory on adipo-commitment, leading to the emergence of adipocytes through IGF1R/FFAR4 cilia-dependent signaling and eventual cilia disassembly (1). In cell culture systems, activation of WNT with WNT3a ligand or pharmacological inhibition of WNT ligands secretion, does not alter number or length of cilia (2). **C** The effects of TGFβ signaling on cilia dynamics exhibit a context- and cell type-specific dependency. Activation of the TGFβ signaling pathway leads to the shortening of primary cilia in cultured chondrocytes (1), whereas it maintains cilia length in cilia of Xenopus left-right organizer (LRO) (2). **D** NOTCH signaling is primarily triggered by the fluid flow-induced alterations in cilia dynamics, which in turn promotes cilia function in diverse cellular systems, such as respiratory epithelium (1), zebrafish left-right organizer (LRO) (2) and endothelial cells in the myocardium (3). This image was created with BioRender.com

Hedgehog (HH) signaling is a prototype of a cilia-dependent pathway decisive for growth and patterning of different tissues and organs during embryogenesis. Key molecular players comprise the transmembrane receptors PTCH, SMO, and GPR161, along with soluble factors including PKA, GLI proteins and the second messenger cAMP [197]. The pathway is induced via HH ligand binding to PTCH and its removal from cilia allowing SMO to enter. Recent studies indicate HH is transported in the neural tube to recipient cells with the help of cytonemes - specialized cellular actin-based extensions that facilitate targeted delivery [198]. SMO accumulation in cilia induces inhibitory receptor GPR161 export by β-arrestin-BBSome-IFT retrograde trains [105]. GPR161 removal decreases cAMP levels and leads to reduced PKA activity [199]. Consequently, GLI3FL (full-length) processing into its repressor form, GLI3R, is inhibited, permitting the transcription of HH-responsive genes. The cilium compartment provides spatial and temporal control over HH signaling, thereby differentiating the effects of ciliary versus non-ciliary cAMP and GPR161 activity [200], [201]. Changes in primary cilia composition induced by HH signaling are rapid and detectable within minutes upon HH pathway activation [202]. Several mechanisms have been proposed to mediate dynamic rearrangements of primary cilia in response to HH pathway activation. HH-mediated SMO activation promotes interactions of GPR161 and PTCH with β-arrestin, which results in the retrieval of both receptors from cilia by endocytosis [203], [204]. Acute HH pathway activation supports primary cilia shortening [125], [205], likely via activation of the ectocytosis/cilia decapitation feedback response [123], [124], [125](Fig. 5A). Certainly, the “heterogeneity/ plasticity” in the timing of the assembly/disassembly of primary cilia noted earlier likely mediates the robustness of HH pathway effects on cell proliferation. In this case, HH pathway activity in either G1-S of the previous cell cycle or the G1 phase of the cell cycle, when the decision is made, is sufficient to drive cell cycle entry [206]. This model is consistent with the remarkable finding that a decision “to divide” could have been made in virtually any phase of the previous cell cycle [207]. HH-induced signaling is thus propagated in a robust manner into the progeny to promote fate decisions within complex tissues.

In muscles, HH signaling promotes the myogenic differentiation of the muscle stem cells [208], at the expense of fibrosis and inflammation [209]. Cilia of these stem cells maintain them in a quiescent state, preserving their ability to generate new myoblasts both during development and in response to stimuli, such as injury [195], [208], [210] (Fig. 5A). During aging, muscle stem cells lose their cilia, causing a decline in regenerative capacity [210]. The primary cilium of muscle stem cells transduces the HH pathway to ensure the proper timing of myoblast differentiation and proliferation [208]. Interestingly, defective cilia formation seems to drive these stem cells into premature proliferation, which can ultimately result in rhabdomyosarcoma [208]. Differentiated myoblasts lose their capacity to assemble primary cilia (hence to respond to HH pathway activation) during myofibrillar transition as they migrate and form myofibers [195]. Regenerative capacity of skeletal muscles is also dependent on fibro/adipogenic progenitors (FAPs) that dynamically modulate their ciliation during muscle regeneration. Preventing FAPs to ciliate blocks adipogenesis and promotes myogenesis following muscle injury [211]. Intriguingly, the mechanism involves the activation of HH target genes, due to the inability of FAPs with ablated primary cilia to generate GLI3R, which otherwise represses the HH target genes [211].

Furthermore, regulation of primary cilia length to fine-tune the responsiveness to HH pathway activation has been proposed to play a key role during the establishment of the dorsolateral axis in the forming neural tube (Fig. 5A). Here, primary cilia of the neural plate HH-responding cells (before the neural tube closure) are relatively short. Intriguingly, following the neural tube closure, HH-producing cells located in the floorplate elongate their cilia and concomitantly attenuate their responsiveness to HH pathway activation [212]. While the attenuation of the HH pathway in the floor plate depends on these elongating cilia, mechanisms driving their elongation and the HH pathway shutdown are still awaiting full determination [212]. One possibility is that the elongation is a consequence of the structural transformation of these cilia to become motile [213]. Cilia remodeling is also occurring during neurogenic divisions of progenitors in the neural tube, in a process termed apical abscission, which physically separates the axoneme and the basal body, hence rendering the cilium temporarily dysfunctional [214]. Remarkably, disrupting the cilium formation during or after its remodeling during the apical abscission impairs axonogenesis in newly born neurons, possibly via a non-canonical HH signaling pathway (not involving regulation of gene expression) [112].

In ciliopathy conditions, such as BBS, cells lack the retrograde adaptor BBSome for retrieval of GPR161 and additional receptors. Activation of the HH pathway in BBSome deficient cells also leads to cilia shortening [125], although the mechanism differs from that in wild-type conditions (Fig. 5A). HH induces hyperactivation here of intraciliary GTPase CDC42, which promotes actin-dependent ectocytosis of the ciliary content, including GPR161, and successively, shortens the ciliary axoneme [125]. Despite these alterations, HH signaling is not entirely suppressed in the BBS context [124], [215]. The GPR161 might likely be removed in ectosomes together with SMO in a bipartite complex [203] or additional HH modifiers, such as PKA [216]. In such cases, the ectocytosis-dependent decrease in cAMP and PKA activity might alleviate GLI3 phosphorylation and sustain low levels of HH signaling.

In Joubert syndrome, upon loss of INPP5E, cilia become shorter and HH signaling increases [123], [217]. INPP5E controls cilium dynamics via regulating ciliary PIP levels and TULP3-dependent import of receptors (e.g. GPR161) to cilia [42], [126]. AURA, as one of the key regulators of primary cilia disassembly, has been depicted to drive cilia shortening via depleting cilia of INPP5E [123]. It remains to be seen if AURA activity also mediates some of the reported effects of the HH pathway on primary cilia length. Overactive HH signaling and short cilia have been also identified in PINK1-deficient human and mouse models of familial Parkinson’s disease [218].

### WNT signaling

Canonical (WNT/β-catenin) and non-canonical WNT (WNT/β-catenin-independent) signaling belong to the major developmental pathways with a somewhat controversial relationship to primary cilium. WNT/β-catenin signaling pathway, similar to the HH signaling pathway, is a potent driver of cell proliferation, with numerous implications for development and cancer [130], [219]. As the name suggests, its key element is the protein β-catenin, which is in response to WNT ligand stimulation translocated from the cytosol into the nucleus, where it, in cooperation with TCF/LEF proteins, drives gene expression [219]. Upon a WNT ligand binding to receptor Frizzled (FZD) and co-receptor LRP5/6, the signal is via activities of scaffolding protein DVL transduced to a complex of APC-AXIN-β-catenin, and kinases such as GSK3β and CK1. This causes the disassembly of this so-called degradation complex, β-catenin escapes GSK3β/CK1-mediated phosphorylation marking it for degradation, which then leads to the stabilization of β-catenin levels and import into the nucleus [219]. The non-canonical pathway is characterized by its β-catenin-independent status and typically involves branches such as WNT/PCP and WNT/Ca^2+^ [130, 220]. Discussion here is focused on the links between WNT/β-catenin signaling and primary cilia, as the connections between cilia and WNT/β-catenin- independent pathways (in particular the WNT/PCP) have been previously identified and thoroughly covered in several reviews [220], [221]. Several components of WNT signaling have been identified associated with basal body/centrosome (e.g. β-catenin, DVL, AXIN, APC) [130] and/or ciliary axoneme (e.g. LRP5/6, FZD) [222]. This evidence alone clearly argues for a link between primary cilia/centrosomes and the components of the WNT/β-catenin signaling pathway. However, functional consequences of such a link for cilia or WNT/β-catenin signaling are considerably less clear. Deregulation of cilia formation/function by acute depletion of some of its regulators (e.g. INVS, BBS1, BBS4, BBS6/MKS, etc.) [223], [224] or mutation of KIF3A in mouse fibroblast[225] leads to concomitant defects in WNT/β-catenin signaling. Conversely, mutation of ciliary regulators in vivo (e.g. IFT88, IFT172, INVS, DYNC2H1, and KIF3A) does not seem to cause major defects in WNT/β-catenin signaling, even though ciliogenesis and/or HH pathway are severely dysregulated [226], [227], [228]. One plausible explanation for these contradicting reports is the activation of compensatory mechanisms that lower the penetrance of the WNT-related phenotypes (in contrast to acute depletion/inhibition by siRNA or morpholino) in models with permanent gene deletion/mutation (e.g. owing to transcriptional adaptation following CRISPR/Cas9 gene editing [229]). Alternatively, some of these effects could be explained by the dual role of some of the cilia components previously discussed (Table 1). Interestingly, compelling evidence suggests that a key step of the WNT/β-catenin cascade, namely the translocation of β-catenin into the nucleus is controlled by IFT-A and kinesin II (KIF3A) proteins. Specifically, lack of IFT140 or KIF3A, though not IFT88, prevents nuclear accumulation of β-catenin following WNT ligand stimulation, independently of their role in primary cilia [150], [151]. Furthermore, reported effects (or their lack of) may reflect the high complexity of the cilia – WNT/ β-catenin signaling relationship (e.g., context-dependent effects, non-cell autonomous functions). The WNT/β-catenin signaling pathway is well established in the specification of motile cilia, via regulation of expression of transcription factor FOXJ, acting upstream of the induction of motile cilia formation [230], [231]. WNT signaling effects on primary cilia dynamics seem to be context-dependent (Fig. 5B). Analysis of cell lines such as hTERT-RPE1, HEK293, and NIH3T3 established that stimulation with WNT3a ligand, pharmacological inhibition of WNT ligands secretion, or genetic ablation of LRP5/6 receptors did not alter the number nor length of primary cilia [232], while ablation or knockdown of LRP5/6 impaired primary cilia formation in HEK293T cells [222]. The explanation may be the cell type-specific activation of a cilia-residing WNT signaling branch, independent of β-catenin and regulation of transcription, yet utilizing LRP5/6 and GSK3β, capable of modulating primary cilia dynamics [222]. The exact mechanism of how the ciliary WNT/GSK3β pathway contributes to cilia dynamics as well as the overall outcome of WNT signaling in ciliopathy conditions remains to be elucidated. In BBS, loss of BBSome stimulates premature cilia disassembly via ectocytosis/cilia decapitation [123], [124], [125]. Simultaneously, it disrupts WNT signaling and leads to altered differentiation of multiple tissues, including the retinal pigment epithelium (RPE) layer in the retina [127], [223], [233], [234]. It is not yet known whether the BBSome regulates the ciliary WNT/GSK pathway. Nevertheless, loss of the BBSome results in dysregulation of the cellular endomembrane system, causing increased exocytosis of the small secretory vesicles containing WNT regulators [235]. An intriguing possibility is that the BBSome may act to balance intraciliary and extraciliary WNT signaling, though this hypothesis needs to be thoroughly tested. It will now be crucial to explore how this WNT/GSK3β signaling route contributes to the overall WNT signaling outcome in ciliopathy models and examine its potential crosstalk with the HH pathway in relevant cases such as cancer and embryogenesis. In fact, considering that dysregulation of either the HH signaling pathway or signaling downstream of LRP5/6 induces neural tube closure defects [220], [236], a developing neural tube could represent a suitable model system to probe for HH pathway alternations in conditions with hampered WNT/(LRP5/6)/GSK3β signaling.

Another system offering room for HH-WNT interactions is mesenchymal cells (Fig. 5B). In mesenchymal cells of the soft palate, WNT/β-catenin signaling has been proposed to facilitate cilium disassembly and proliferation during myogenesis [237]. The WNT signaling is here likely accompanied by the HH pathway activation, which is essential for the proper myogenic differentiation as well [208, 210]. WNT and HH pathways are vital for driving the osteo-lineage commitment of mesenchymal stem cells (MSCs) during bone growth and upon mechanical loading [238, 239], [240]. Loss of IFT20 skews the osteo-/adipo-commitment of MSCs toward the adipogenic lineage [239], emphasizing the critical role of functional cilia in this process while still leaving open the possibility for IFT20 extraciliary functions. In contrast, the commitment of MSCs to adipogenic lineage is suppressed by WNT and HH pathways and driven by the IGF-1R and FFAR4 signaling [241], [242]. The primary cilium here is present only on committed pre-adipocytes to secure the onset of the adipogenic program and then disappears in mature adipocytes [242, 243], [244]. WNT signaling in MSCs also regulates the production of chemokines, such as CXCL12, being essential to maintaining hematopoiesis in bone marrow [245]. BBS patients and mouse models exhibit altered blood parameters, with a notable decrease in CXCL12 production observed in the bone marrow of BBS mouse models [246]. This indicates that ciliary factors, IFT20, BBS4, and BBS18 in MSCs regulate both the intrinsic and extrinsic functions of MSCs in bone tissue. However, it remains unclear whether BBSome deficiency influences the production of extracellular vesicles in MSCs, as seen in cell lines [235], and how this, consequently, impacts bone marrow homeostasis.

### TGFβ signaling

The TGFβ signaling pathway regulates a wide range of cellular processes, including cell proliferation and differentiation. Upon stimulation with the TGFβ ligand, the cilia localized receptors TGFβIR and TGFβIIR become internalized via the clathrin-mediated endocytosis [247], [248]. Ciliary pocket (CiPo) is considered prominently decisive in TGFβ receptor retrieval, hence acting upstream of the phosphorylation of key effector proteins, SMAD2/3, to promote their transport into the nucleus to drive gene expression (reviewed in greater detail in [249]). Noteworthy, the involvement of CiPo in clathrin-mediated endocytosis of TGFβ receptors in vertebrates is pursuant to the function of the analogous region, the flagellar pocket, which serves as the sole site of endocytosis in protists [39]. A defect in TGFβ signaling has been reported following the ablation of IFT88 and CEP128 (a component of SDAs), causing defects in dorsoventral patterning in early embryogenesis [247], [248]. Surprisingly, depletion of CEP128 impairs ciliary TGFβ signaling while leaving primary cilia largely intact (in fact, its depletion facilitates cilia formation and promotes their length), leaving the contributing mechanism expected to hamper receptor trafficking at CiPo to be found.

Similar to the WNT signaling pathway, the effects of TGFβ signaling on cilia dynamics are context- and cell type-dependent (Fig. 5C). While activation of the TGFβ signaling pathway triggers shortening of primary cilia in cultured chondrocytes [250], cilia of Xenopus left-right organizer and other tissues reveal reduced length following the blocking of TGFβ pathway [251], and cilia remain largely intact in human mesenchymal stem cells in response to deregulated TGFβ signaling [252]. Underlying mechanisms involve the regulation of gene expression of key regulators of cilia assembly. Specifically, TGFβ signaling suppresses IFT88 expression in chondrocytic cells, resulting in a reduction in both the number and length of cilia [250]. Conversely, TGFβ signaling in Xenopus is required to sustain the expression of TZ components, and hence cilia maintenance [251].

Aberrant TGFβ signaling is linked to congenital heart diseases and skeletal anomalies, which are common features of ciliopathies and has been recently associated with Alstrom syndrome [223], [224], [225]. In mesenchymal stem cells, TGFβ signaling drives the differentiation of myogenic, chondrogenic, and adipogenic lineages [239], [253], [254]. Loss of IFT20 decreases TGFβ driven SMAD2/3 phosphorylation, impairs glucose metabolic homeostasis, and promotes adipolineage commitment of the bone marrow MSCs [239]. TGFβ signaling is critical for bone regeneration during fracture healing by regulating the expression of angiogenic and chondrogenic genes in chondrocytes and recruitment of the bone marrow MSCs to the site of injury [252], [253].

Decrease in primary cilia frequency or length upon loss of IFTs or by upregulation of cilia disassembly factors can be also linked to altered TGFβ-mediated extracellular matrix protein expression, which is a common prerequisite of fibrosis and cancer in many tissues, such as atrial fibrosis [254], lung pulmonary fibrosis [255], polycystic liver disease [256], liver fibrosis [257], cholangiocarcinoma [258], or colon cancer [259].

### NOTCH signaling

NOTCH signaling is a developmental pathway controlling intercellular communication during tissue morphogenesis [260], its correct regulation relying on functional basal body/cilium, for instance, in developing skin [261] or in liver [262]. NOTCH signaling is transduced by the interaction of two cells, where one cell provides one of the DELTA-like (DLL1, DLL3, and DLL4) or JAGGED ligands (JAGGED1, JAGGED2) and the second one the receptors (NOTCH 1–4). Ligand–receptor interaction triggers γ-secretase-dependent cleavage of the NOTCH intracellular domain (NICD), which then translocates from the cytoplasm to the nucleus, where it promotes expression of key transcription factors *Hes* and *Hey* regulating the expression of downstream genes [263]. NOTCH pathway components, including the NOTCH receptor, were detected at the basal body and/or ciliary axoneme [163, 264]. The spatial organization of NOTCH signaling is regulated by the polarized trafficking module involving ARF4-RAB11 GTPases [264], [265]. Once at the membrane, the NOTCH receptor is continuously recycled through the endomembrane system back to the surface to maintain signaling capacity. Loss of BBS1, BBS4, or ALMS results in overactivation of the NOTCH pathway, likely owing to NOTCH retention in sorting endosomes [163].

Cilia-deficient keratinocytes in vitro and in mice embryonic epidermis have defects in NOTCH signaling [261]. Similarly, cKO of IFT88 leads to an increased proliferation yet decreased NOTCH signaling in corneal epithelial cells [266]. Furthermore, ablation of cilia-localized PKD2 in the liver induces increased NOTCH signaling and formation of cysts [262]. The primary cilium is also needed for hematogenic endothelial cell (HE) differentiation through the regulation of NOTCH signaling. HE cells give rise to hematopoietic stem and progenitor cells (HSPC). Reduced expression of HSPC markers, RUNX1 and CYMB, was observed in cilia-impaired zebrafish embryos due to reduced protein levels of NICD [267].

Studies examining the relationship between cilia dynamics and NOTCH signaling primarily focus on fluid flow-induced changes in cilia length and signal transduction, revealing an apparent interdependence between these two processes (Fig. 5D). Hyper-activation of the NOTCH pathway by overexpressing the NCID or DELTA-like ligand results in prolongation of cilia in the left-right organizer in zebrafish embryo. Here, the fluid flow-induced change in the cilium length triggered NOTCH signaling for transcriptional activation of laterality genes [268]. Similarly, in the endocardium, the mechanical shear stress signals via cilia activate the NOTCH pathway and regulate regeneration in zebrafish hearts [269]. During airway differentiation, NOTCH activity regulates levels of PROM1, a stem cell marker, to establish multiciliated cell diversity in the respiratory tract and modulate mucociliary clearance [270]. High NOTCH and PROM1 levels produce multiciliated cells with fast-beating cilia, whereas low levels result in cells with longer, low-beating cilia. It will be interesting to investigate whether increased mucociliary shear stress, induced e.g. by infections, increases cilia beating and further NOTCH activation similar to the shear stress in the endocardium or the fluid flow in the LRO [268], [269].

While not covered in this review, additional regulatory signaling circuits are involved in ciliogenesis control, including the tyrosine kinase signaling receptor, and HIPPO -YAP/TAZ pathway. Several excellent reviews concerning ciliogenesis control circuits may be referenced here [249], [271].

## Managing cilia length and function: therapeutic strategies for converging mechanisms

Disruptions in ciliary dynamics are implicated in both ciliopathies and cancer progression, underscoring the therapeutic potential of targeting cilium-related processes. On one hand, strategies aimed at restoring cilium length and function show considerable promise in reducing cancer cell division and tumor growth [255], [258]. On the other hand, stabilization of the primary cilium helps cancer cells adopt a quiescent, stem-like state, which shields them from treatments based on kinase inhibitors [272]. In this part, we highlight small-molecule compounds that effectively modulate specific pathological mechanisms, including the regulation (both upregulation and downregulation) of the Hedgehog signaling pathway and the assembly and disassembly of primary cilia - processes closely associated with cancer development. Some of these inhibitors are already approved for specific conditions, and their use, either alone or in combination, holds the potential for addressing a broader range of cilia-associated disorders.

### Targeting the HH signaling pathway

A key characteristic of cilium dysfunction is the disruption of signaling pathways, such as HH, which is commonly observed in conditions like Joubert syndrome, polycystic kidney disease, and Bardet-Biedl syndrome [5]. SMO inhibitors, such as cyclopamine or purmorphamine, have demonstrated potential in regulating excessive HH signaling caused by shortened or malformed cilia. This has been observed in conditions like Parkinson`s disease [218], in nephronophthisis associated with CEP290 mutations in Joubert syndrome [273], [274], and abnormal brain ventralization linked to dysfunctional INPP5E [217].

Increased HH signaling is also linked to the development of basal cell carcinoma and medulloblastoma [275], [276]. Here, the tumor growth can be driven by hyperactivated SMO in the presence of primary cilium and also by active GLI2, independent of the primary cilium. This indicates that the role of primary cilium is determined by the specific oncogene driving HH-triggered cancers, emphasizing the need for accurate diagnostics to select the most effective compounds or HH inhibitors to target pathogenic HH signaling [275], [276], [277]. In the case of brain tumors dependent on cilia, employment of TTBK1/2 inhibitors could represent a promising strategy to mitigate cilia-dependent HH upregulation [278], [279].

Activation of HH signaling plays a critical role in osteoarthritis [280] and can be suppressed by LiCl-mediated cilia prolongation in chondrocytes [281]. Similarly, LiCl treatment inhibits HH signaling in pancreatic ductal carcinoma cells leading to suppression of their tumorigenic phenotype [282]. Since LiCl is widely used for the treatment of people with bipolar disorders, it remains to be elucidated whether LiCl modulates the neuronal ciliary signaling pathway involving GPCRs, such as the dopamine receptor. Disrupted ciliary signaling, especially the HH pathway, could therefore serve as an ideal therapeutic target for ciliopathies, neurodegenerative disorders, and cancers driven by its dysfunction or exploitation.

### Targeting the cilia disassembly factors HDAC6 and AURA

As previously noted, the mechanisms of the AURA/HDAC6 module in regulation cilia dynamics leave several questions unanswered. Importantly, however, strategies based on small molecules targeting those regulators seem to restore cilia homeostasis in several cilia dysfunction-related pathologies. In conditions like BBS and renal anomalies, including polycystic kidney disease and nephronophthisis, defects in cilia dynamics were mitigated using HDAC6 inhibitors [283], [284], such as tubacin [127], ACY-1215 [285] or tubastatin [286]. Similarly, in Rett syndrome, mutations in the MECP2 gene lead to microtubule instability, and tubacin treatment restored impaired HH signaling [287].

Another phenotype associated with ciliopathies like Bardet-Biedl, Alstrom, and MORM syndromes, is obesity [8]. Obese adipose-derived MSCs possess dysfunctional cilia with compromised HH signaling [288]. Notably, treatment with HDAC6 or AURA inhibitors, such as MLN8054, restored cilium length and expression of genes associated with osteogenic and adipogenic lineage commitment in these cells [244], [288]. This is in agreement with the view that primary cilia maintain stem cells in a quiescent state while preserving their capacity to differentiate, as noted earlier [208], [210], [239], [244].

HDAC6 inhibitors are also effective in cancer therapies, as many cancer cells rely on deregulated cilia dynamics for rapid cell division. In cholangiocytes, overexpression of HDAC6 drives increased cell proliferation, contributing to cystogenesis and tumor progression [258]. Thus, by stabilizing cilia and disrupting the timing of ciliary disassembly needed for mitotic entry, these inhibitors impair cancer cell proliferation. Furthermore, combining HDAC6 or AURA inhibitors with inhibitors of other pathways overactive in cancer cells, such as WNT or GFR (growth factor receptor) signaling [255], can more effectively halt cancer progression and potentially overcome resistance to single-pathway inhibitors.

Altogether, numerous studies underscore the importance of stabilizing cilia not only to address ciliopathies and cancer but also to preserve the regenerative potential of tissues. This highlights the therapeutic promise of HDAC6 and AURA inhibitors, which modulate ciliary dynamics and help restore normal cellular signaling in these conditions. An advantage of HDAC6 inhibitors is they are non-toxic to healthy tissues, and HDAC6-deficient mice do not exhibit any pathological phenotype under normal conditions [116]. However, mutations in HDAC6 lead to an increase in axoneme glycylation [117], suggesting that HDAC6 inhibitors may similarly activate compensatory mechanisms to preserve axoneme stability. HDAC6 is part of a large family, and certain HDAC6-selective inhibitors lack specificity, potentially causing off-target effects. Furthermore, HDACs are involved in various cellular processes, and off-target effects could lead to unexpected adverse phenotypes. Further research is therefore necessary to design more selective and effective HDAC6 inhibitors.

### Therapeutical potential in compensatory mechanisms of ciliary modules

Understanding the mechanisms by which ciliary modules regulate cilia dynamics opens new avenues for developing new strategies to restore cilia function in ciliopathies. In BBS, cilia shortening is promoted by increased F-actin polymerization [125], [289] and elevated PIP2 within cilia [289]. Accumulation of PIP2 and F-actin in cilia phenocopies the loss of function INPP5E phenotype in Joubert syndrome [123]. Here, overexpressing INPP5E in olfactory neurons lacking BBSome restored cilia length and rescued odor detection and perception in BBS mice [289]. Another strategy to restore cilia length in BBS could involve suppressing the overactivated ciliary CDC42, thereby reducing excessive intraciliary actin polymerization and preventing ectocytosis to stabilize and elongate cilia [125]. One might potentially aim to target cargo import regulation [97], [144] to reduce the accumulation of GPCRs (SMO, GPR161, etc.) in cilia of BBSome-deficient cells, mitigating ectocytosis and overall cilia destabilization.

## Challenges and future directions

Addressing shared mechanisms offers considerable potential for developing therapies applicable across multiple disorders, creating a framework for targeted and tailored treatment strategies. Combining inhibitors of ciliary disassembly, such as HDAC6 or AURA inhibitors, with approaches to modulate the activity of INPP5E, CDC42, or cargo import, could synergistically enhance cilia stabilization. Reducing actin polymerization might, for example, improve the effectiveness of disassembly inhibitors by mitigating another destabilizing force within cilia. By focusing on both structural (axonemal and F-actin dynamics) and functional (cargo trafficking and signaling) aspects of ciliary maintenance, these approaches offer a dual mechanism to restore cilia length and function, particularly in diseases where multiple pathways are disrupted.

## Data Availability

Not applicable.
